# Mixture of experts for multitask learning in cardiotoxicity assessment

**DOI:** 10.1186/s13321-025-01072-7

**Published:** 2025-08-29

**Authors:** Edoardo Luca Viganò, Mateusz Iwan, Erika Colombo, Davide Ballabio, Alessandra Roncaglioni

**Affiliations:** 1 Laboratory of Environmental Toxicology and Chemistry, Department of Environmental Health Sciences, Instituto Di Ricerche Farmacologiche Mario Negri IRCSS, 20156 Milan, Italy; 2 Milano Chemometrics and QSAR Research Group, Department of Earth and Environmental Sciences, University of Milano-Bicocca, 20126 Milan, Italy

**Keywords:** Artificial Intelligence, Multitask Neural Network, Cardiotoxicity, Machine Learning, QSAR, Mixture of Experts

## Abstract

**Abstract:**

In recent years, the integration of Artificial Intelligence and Machine Learning methods with biochemical and biomedical research has revolutionized the field of toxicology, significantly advancing our understanding of the toxicological effects of chemicals on biological systems. Cardiovascular diseases remain the leading global cause of death. The constant exposure to multiple chemicals with potential cardiotoxic effects, including environmental contaminants, pesticides, food additives, and drugs, can significantly contribute to these adverse health outcomes. Traditional methods for assessing chemical hazards and their impact on biological function heavily rely on experimental assays and animal studies, which are often time-consuming, resource-intensive, and limited in scalability. To overcome these limitations in silico methods have emerged as indispensable tools in toxicological research, reducing the need for traditional in vivo testing and conserving valuable resources in terms of time and cost. In this study, Artificial Intelligence methods are used as first-tier components within an Integrated Approach to Testing and Assessment. We explored the potential benefits of using Multitask Neural Networks, where multiple levels of cardiotoxicity information are combined to enhance model performance. Multitask learning, based on specific architectures such as Mixture of Experts (MoE), showed promising results and surpasses the performance of single-task baseline models. When predicting a holdout set, multitask model achieved high performance on twelve different endpoints related to cardiotoxicity defined by Adverse Outcome Pathways Network. The best developed model achieved a balanced accuracy of 78%, a sensitivity of 80%, and a specificity of 76% across all endpoints in the holdout set.

**Scientific contribution:**

An advanced multitask model was developed to predict cardiotoxicity mechanisms induced by small molecules. The model demonstrates broad mechanistic coverage and achieves performance comparable to, or exceeding, state-of-the-art methods. These results suggest that the model could serve as a valuable first-tier component in advanced New Approach Methodologies for prioritizing chemicals for further testing.

**Supplementary Information:**

The online version contains supplementary material available at 10.1186/s13321-025-01072-7.

## Introduction

Cardiovascular diseases (CVDs) are the leading cause of mortality worldwide [1–5]. The exact reasons behind cardiovascular disorders are not always clear, as they involve a variety of risk factors and mechanisms influenced by lifestyle and environmental conditions. As a result, accurately predicting the onset or progression of cardiovascular diseases can be very challenging. Several classes of chemicals, including drugs, pesticides, environmental pollutants, industrial agents, and food additives can further contribute to the observed health effects upon exposure [2, 5–8]. This issue, however, remains poorly addressed and regulated [3, 6].

The in-silico models developed in our study are designed to serve as first-tier components within an Integrated Approach to Testing and Assessment (IATA) [9]. This framework incorporates New Approach Methodologies (NAMs) to support Next-Generation Risk Assessment (NGRA). By using in-silico models, the testing of chemicals may be limited to cases where uncertainties from lower-tier evaluations are unacceptable, before performing resource-intensive laboratory testing or animal studies. In this study, we modelled the potential hazards of compounds following the concept of Quantitative Structure–Activity Relationships (QSARs). QSAR is a computational approach that uses the chemical structure and other relevant properties of a molecule to predict its biological activity. In-silico models based on this approach learn how the properties of a molecule are related to its activity [10–12]. Since cardiotoxicity is complex and broad term, it is crucial to carefully define the evaluated endpoints. The OECD Guidance Document on the Validation of QSAR Models emphasizes that a well-defined endpoint is fundamental for reliable modeling [13]. Indeed, a clear and transparent definition of the predicted endpoint is essential for regulatory assessment and ensures the validity of predictions for specific applications. Additionally, the experimental protocol—including study design, laboratory procedures, assay acceptance, and evaluation criteria—plays a critical role in data quality and endpoint definition. For these reasons in this work, we considered only chemicals that were tested using precise protocols, passed quality control, and were relevant to our study. The relevant toxicity effects were selected based on the Adverse Outcome Pathways (AOP) framework for cardiotoxicity [6–8, 14, 15]. AOPs are a framework for organizing and presenting scientific knowledge on how a stressor (e.g., a chemical) impacts a biological target, pathway, or process, leading to adverse outcomes relevant to risk assessment, regulation, and environmental management. These networks are built of standardized characterizations of molecular initiating events (MIEs) and other biological key events (KEs) ultimately leading to a regulatory relevant adverse outcome (AO) [16]. In our work, we collected data on MIEs, KEs, chemical-specific Modes of Action (MOAs) and apical cardiotoxicity effects (which are CVDs-related drug side effects collected in FDA reports) aimed to assemble an extensive dataset that evaluated drug-induced cardiotoxicity on multiple levels.

Notably, in the literature, there is a wide range of QSAR models applied to predict toxicological endpoints related to cardiotoxicity. Among them, hERG channel inhibitors are extensively studied, with a substantial number of data, providing the opportunity to develop high-performance QSAR models [17–24]. Additionally, mitochondrial dysfunction, Aryl Hydrocarbon Receptor (AhR) inhibition, and increased oxidative stress are other MIEs and/or KEs that have been widely explored and modeled, as they can lead to various toxic effects beyond cardiotoxicity, such as steatosis and cholestasis [25–30]. However, the majority of models in the literature are highly specialized in predicting individual toxicity effects, which limits their applicability and reduces their generalization capability. Also treating each biological interaction separately limits the ability of Artificial Intelligence (AI) approaches to fully capture the underlying mechanisms driving these interactions, reducing their performance. Furthermore, assessing chemicals using multiple in-silico models to cover a broad spectrum of endpoints related to cardiotoxicity can be very challenging and time-consuming.

To address these limitations, this work focuses on developing a multitask neural network (MTNN) capable of predicting multiple cardiotoxicity effects simultaneously with a single model. We prioritized Multitask Learning (MTL) because it has shown promising results in the field of toxicology [31–35] and is particularly relevant in cases like ours, where chemicals are tested across multiple biological cascade effects that may lead to the same final adverse outcomes. Furthermore, several advantages of using MTL can be found in the literature [31, 32, 34]. This approach enables the use of a broader pool of data, improves the model’s learning process and expands its applicability domain.

**Fig. 1:**
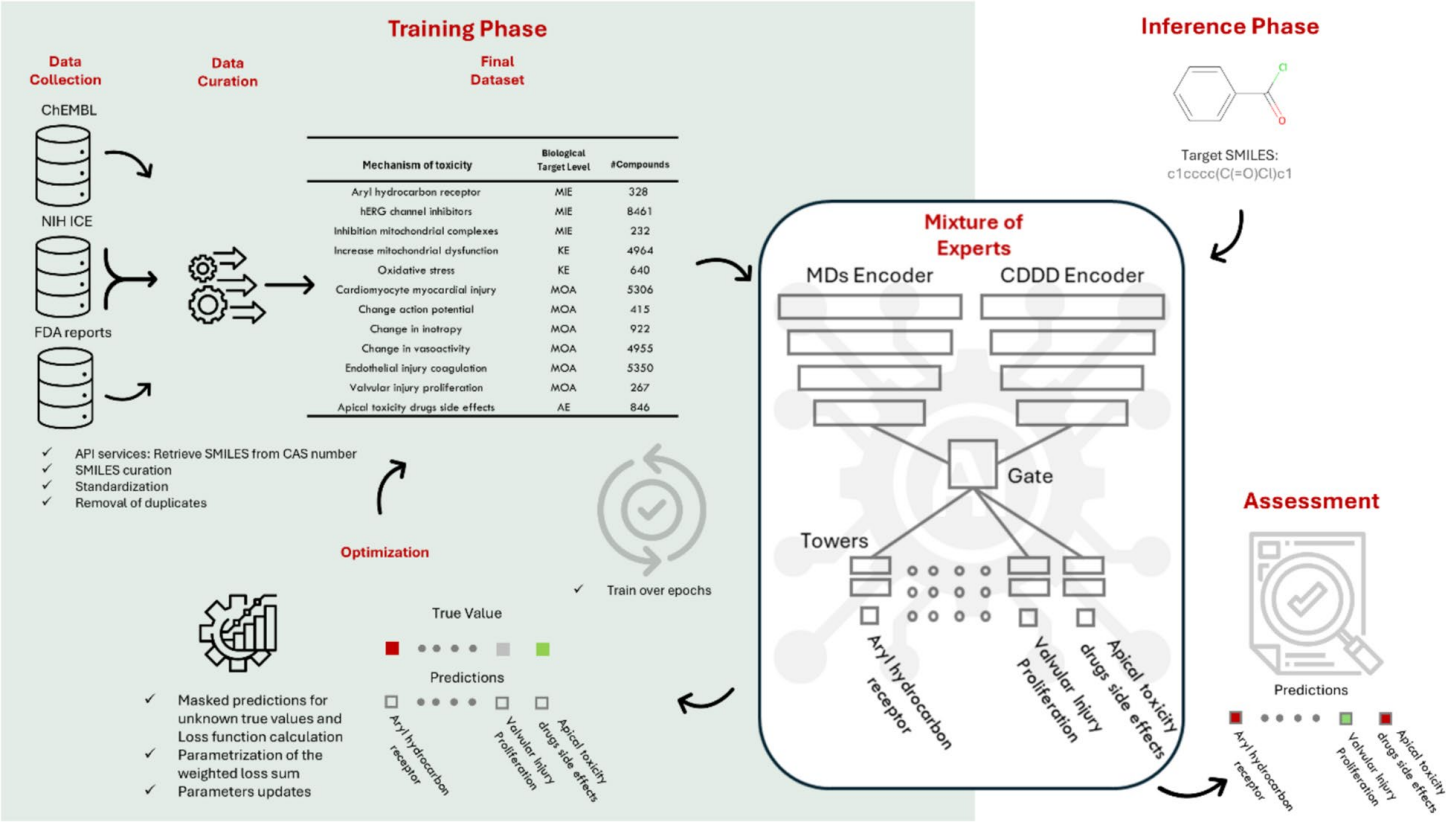
The training phase (green panel) integrates data from ChEMBL, NIH ICE, and FDA reports, followed by curation steps such as SMILES standardization, duplicate removal, and biological target annotation. The curated dataset, which includes mechanisms of toxicity across different biological target levels, is used to train the model. The chemical information for each compound is encoded using different methods that serve as inputs to the model. The model, composed of multiple branches and task-specific towers, is trained through an optimization process guided by masked loss calculations. In the inference phase (right panel), a target SMILES is input into the trained model, and predictions are generated for the toxicological endpoints

In Fig. 1, we present a flowchart illustrating how our model is trained and how it performs inference to assess the target molecule.

Additionally, MTL serves as a form of inductive transfer, enhancing modeling outcomes by introducing an inductive bias. This bias encourages the model to favor certain hypotheses over others by leveraging auxiliary tasks (which are single biological effects in our case) to guide the learning process. As a result, the model considers the hypotheses that explain multiple endpoints simultaneously, often leading to better generalization. By sharing information across tasks, the multitask model can predict each endpoint more effectively than single-task models, thereby overcoming their inherent limitations.

## Materials and methods

### Dataset

The data used in this study were collected from three main sources: the NIH ICE database, ChEMBL [36, 37] and documents related to FDA (U.S. Food and Drug Administration)-approved drugs [38]. The latter includes the DICTrank dataset, which, as claimed by its authors, represents the largest collection of drugs annotated with ranked drug-induced clinical toxicity (DICT) risk in humans. The ICE database provides curated data intended to support the development and evaluation of new, revised, and alternative methods and is developed by NICEATM, ICCVAM and their partners. ChEMBL is a manually curated database for small, drug-like molecules that includes information about their structures, molecular properties, measurements from various assays, and genomic data.

The chemicals were retrieved using CAS numbers, which are unique identifiers assigned to each molecule. To obtain the Simplified Molecular Input Line Entry System (SMILES), we used an in-house software (https://github.com/EdoardoVigano/Chemical-Resolver), which can query the API service of reliable databases such as ChEMBL and PubChem. The obtained SMILES strings were then standardized following a protocol [39, 40] that involves removing organometallic and inorganic compounds, eliminating chemicals with structural inconsistencies and mixtures, and removing the stereochemistry information. The SMILES were then canonized, and duplicate structures were removed.

Each chemical was then labeled as active for a given biological target if at least one of the assays for that target had a positive label, and inactive otherwise. To determine positivity in a test, we followed the procedure described in the *User's Guide for Accessing and Interpreting ToxCast™* [41]. Using this approach, we compiled a final dataset consisting of 14,688 unique chemicals, each annotated with binary labels (active or inactive) for 12 different toxicity endpoints, as summarized in Table 1. Notably, the dataset is imbalanced across most endpoints.

**Table 1:** Summary of data collection, including the total number of chemicals and the percentage of active and inactive compounds

	Biological target level	Total no. of compounds	No. of active	No. of inactive	Active%	Inactive%	Source
Aryl hydrocarbon receptor	MIE	328	211	117	64	36	ChEMBL [36]
hERG channels inhibitors	MIE	8461	4294	4167	51	49	ChEMBL [36]
Inhibition mitochondrial complexes	MIE	232	184	48	79	21	ChEMBL [36]
Increase mitochondrial dysfunction	KE	4964	1131	3833	23	77	NIH ICE [37]
Oxidative Stress	KE	640	193	447	30	70	NIH ICE [37]
Cardiomyocyte Myocardial Injury	MOA	5306	1558	3748	29	71	NIH ICE [37]
Change Action Potential	MOA	415	111	304	27	73	NIH ICE [37]
Change in Inotropy	MOA	922	213	709	23	77	NIH ICE [37]
Change In Vasoactivity	MOA	4955	1009	3946	20	80	NIH ICE [37]
Endothelial injury coagulation	MOA	5350	2290	3060	43	57	NIH ICE [37]
Valvular Injury Proliferation	MOA	267	91	176	34	66	NIH ICE [37]
Apical toxicity drugs side effects	Adverse Effect	846	620	226	73	27	FDA report [38]

Specific details about the data sources and retrieved data along with statistics on dataset composition are provided in Additional file 1: Sect. 1

### Molecular representations

Numerous methods suitable for encoding chemical information in a machine-readable format can be found in the literature [39, 42–45]. However, the efficacy of each method depends on the endpoint. To find the most suitable encoding for our task, we evaluated Morgan Fingerprints [46], Molecular Descriptors from the Mordred software [43], CDDD descriptors [39], and ChemBERTa embeddings [36].

Morgan Fingerprints are fixed-length, binary representations. They are obtained by first assigning atom numbering-independent values to all heavy atoms within a molecule and iteratively updating them based on the neighborhood of each atom. When subsequent iterations do not add new information, or if the pre-defined fragment size limit is reached, a hash function is used to convert the obtained set of values into final vector. We used the standard values of 1024 bits and a radius of 2 and calculated the fingerprints using Rdkit (version 2023.09.4).

Molecular descriptors (MDs) describe each compound using thousands of numerical values representing different chemical properties, such as polarizability, steric hindrance, molecule shape, etc. In other words, MDs are quantitative representations of chemicals, capturing various aspects of their chemical structure, properties, or behavior. We calculated the MDs using Mordred [43], obtaining 1613 different one- or two-dimensional descriptors.

CDDD descriptors are derived from an encoder-decoder model trained to encode SMILES strings. The encoder transforms SMILES strings into a numerical vector—called a latent representation—while preserving grammatical, semantic, and chemical information [39]. Additionally, the compact size of this representation facilitates the handling of complex chemical structures. The latent representation can then be used for various tasks, including similarity analysis, clustering, and property prediction.

ChemBERTa is a natural language processing (NLP) model tailored for chemistry tasks [45]. It adapts the BERT (Bidirectional Encoder Representations from Transformers) architecture, originally developed for text-based NLP tasks, to work with SMILES strings ChemBERTa bridges the gap between advanced NLP techniques and cheminformatics, enabling the analysis of chemical data using state-of-the-art machine learning approaches.

### Baseline models

We developed a Random Forest (RF) model for each combination of endpoint and encoder, resulting in four RF models for each of the MOAs, MIEs, KEs, and apical cardiotoxicity endpoints. The models used the following encoders: ChemBERTa embeddings, Morgan fingerprints, molecular descriptors, and CDDD. For each task, data were split into a training set (80%) and a holdout set (20%), stratified by label as defined in Sect. “Internal and external validation”. The same split was used for both the baseline and multitask models. A grid search was conducted to optimize hyperparameters, considering the number of trees, maximum depth, and minimum samples per leaf. The F1-score was used as the evaluation metric during hyperparameter tuning, which is made perform 5-fold stratify cross-validation on the training data for each of the parameter’s combinations and use the median of the obtained value. This helps in getting an unbiased estimate of model performance. After selecting the optimal hyperparameters, RF models were externally evaluated on the holdout set.

### Deep neural networks architectures for multitask learning

A Multitask Neural Network (MTNN) is a deep learning model designed to learn multiple related tasks simultaneously using a shared architecture. A typical MTNN architecture consists of shared backbone layers that extract features, followed by task-specific layers. This approach allows the model to leverage shared representations, improving generalization and reducing overfitting. Training is performed using a joint loss function that balances performance across tasks.

MTNNs are increasingly being used in QSAR and toxicology, as they allow the integration of multiple data sources and can improve model performance [31, 33, 34, 47].

**Fig. 2:**
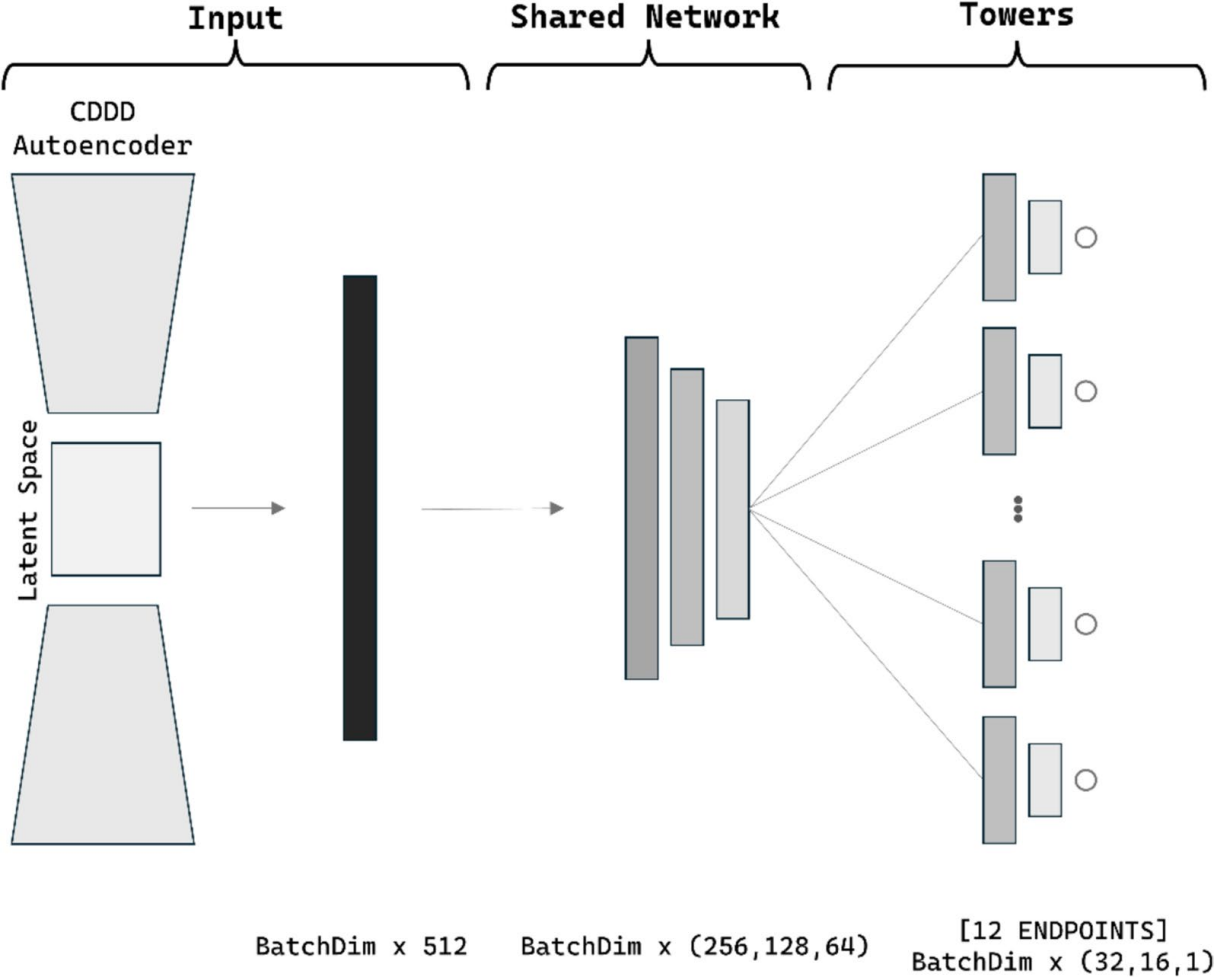
Common architecture used across multiple encoders. List of layers and visual representation of the architecture is reported

We conducted multiple tests to explore various model architectures for MTL, using grid search to identify the optimal layer configuration. We also evaluated different activation and loss functions, aiming to achieve high performance in binary classification for cardiotoxicity endpoints. We explored the optimal architecture for each of the encoders under evaluation, which, include CDDD, MDs, ChemBERTa, and Morgan fingerprints, as previously mentioned. Since the optimal architecture varied slightly across cases, we prioritized simplicity in our study, by using the same architecture for all single-encoder networks. Only the input layers were modified, while the rest of the architecture remained consistent (see Fig. 2).

This approach is fundamental to our aims, as it enables a direct comparison between the encoders using the same network and number of parameters except for the input layer. This provides the possibility to determine whether one encoder is particularly suitable for our case study and, if so, which is the most effective one to maximize model performance.

To address the shortcoming of NLP-based methods—such as focusing on only parts of SMILES strings and lacking information about the characteristics of entire molecules—we explored model architectures in which separate branches use different molecular representations to obtain more comprehensive information about chemicals. Further details about encoder architecture for NLP branch and gate layer are reported in Additional file 1: Sect. 3.

**Fig. 3:**
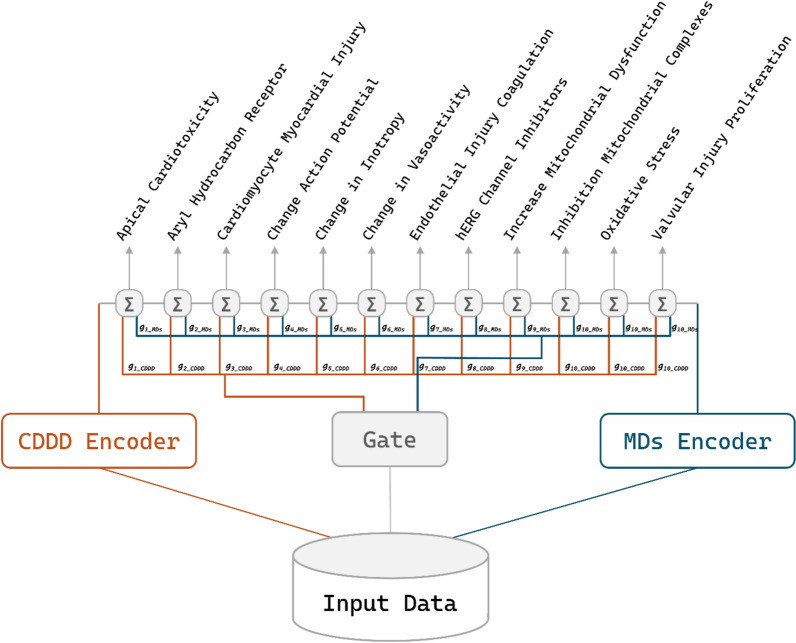
The MoE architecture is presented as a simplified schema, where the integration of experts is weighted by the gate

In a MoE architecture, information from multiple sources is considered and weighted by the gating mechanism of the architecture. These weights, which represent the relative importance of information provided by the encoders, are network parameters that the model learns during training. The architecture of MoE with two branches used in this work is reported in Fig. 3.

The gate layer is particularly important, as it enables the development of a sort of attention-mechanism specifically focused on the encoders. To extend our study, we explored a MoE using all encoders from the baseline models and compared our manual encoder selection with the gating mechanism’s weighting. Using all available data, we conducted 10-fold cross-validation, rebuilding the model each time, updating the gate values, and reporting the mean with a 95% confidence interval (*t*-student test, alpha = 0.05).

### Internal and external validation

The dataset is split into a training set, validation set, and holdout set, with stratification by label for each task under evaluation, with a split ratio of 8:1:1. Since we had multiple endpoints, we began by stratifying the less numerous datasets and then concatenating the chemicals that are not already present from the more numerous datasets. With this approach, we can maintain the stratification for each endpoint as much as possible.

Notably, since the validation set is not usable for the baseline model, we decided to merge it with the training set. This can be considered an advantage for machine learning, as the models ultimately benefit from a larger training dataset compared to Multitask approaches.

The baseline models, developed after hyperparameter tuning, are evaluated internally through 5-fold cross-validation and externally by assessing their performance on the holdout set.

Regarding the deep learning architecture for the multitask model, the internal validation workflow follows the same conceptual approach as that used for the ML model. The model is retrained 10 times on different data splits for multiple epochs. Each epoch consists of a forward pass and a backward pass for all samples. During each epoch, the input data is processed through the model to generate predictions, and the model parameters are updated based on the prediction errors evaluated on the validation set. This iterative process allows us to track the history of the selected optimization metric as it converges to a minimum over the epochs.

During the training phase, the models aim to minimize the loss function defined in Sect. “Loss function and model training”. Once cross-validation is completed and model stability is demonstrated across different folds, the final model is trained using fixed training, validation, and holdout split, consistent with the one used for the baseline models.

### Loss function and model training

We tested two different approaches based on the weighted Binary Cross-Entropy (BCE) for calculating the loss during training. In the first one, the loss is calculated simply as a means of losses for each task, after applying a sigmoid layer and masking missing values. The formula for calculating the loss func

(1) $${\text{l}}\left( {\text{x,y}} \right) = {\text{mean}}\left( L \right) = \left\{ {{\text{l}}_{1} , \ldots , {\text{l}}_{{\text{N}}} } \right\}^{{\text{T}}} ,\,{\text{l}}_{{\text{n}}} = - \left[ {{\text{y}}_{{\text{n}}} \cdot \log\upsigma \left( {{\text{x}}_{{\text{n}}} } \right) + \left( {1 - {\text{y}}_{{\text{n}}} } \right) \cdot \log \left( {1 -\upsigma \left( {{\text{x}}_{{\text{n}}} } \right)} \right)} \right]$$

where ${\text{y}}_{{\text{n}}} { }$, is the true value of the nth task, ${\text{x}}_{{\text{n }}}$, is the prediction for the nth task, $\upsigma$, sigmoid function.

Considering a weighted sum instead of a simple mean should be considered improvement, as it could account for different number of entries per endpoint and for differences in the variance of each dataset. The weights could be, for example, found using a systematic grid search. This approach would, however, require substantial computational resources, without guaranteeing finding the optimal weights. Following an approach described by Kendall in [47], we introduced additional, learnable parameters to the model that would serve as summing weights for individual tasks while accounting for the variability inherent to datasets used for training (i.e. homoscedastic aleatoric uncertainty). Indeed, this approach is based on the hypothesis that a multi-task model can achieve higher accuracy by using a loss function that weights each task according to the uncertainty associated with data variability, rather than relying on fixed weights. Uncertainty-based weighting is an effective method for dynamically balancing the contributions of multiple tasks in a multitask learning setup.

The formula for calculating the loss function with the additional weighting parameters is presented in Eq. (2).

(2) $${\text{Loss}}({\text{W}}, \upsigma _{1} , \ldots , \upsigma _{N} , {\text{y}}), {\text{f}}\left( {\text{x}} \right) = \mathop \sum \limits_{{{\text{i}} = 1}}^{{\text{N = task}}} \frac{1}{{\upsigma _{{\text{i}}}^{2} }} \cdot {\text{BCE}}\left( {{\text{y}}_{{\text{i}}} , {\text{f}}\left( {\text{x}} \right)_{{\text{i}}} } \right) + \mathop \sum \limits_{{{\text{i}} = 1}}^{{\text{n}}} \log \left( {\upsigma _{{\text{i}}} } \right)$$

where W, model parameters; $\upsigma _{{\text{i}}}$, A trainable parameter representing the uncertainty of task ${\text{y}}_{{\text{n}}} { }$, ground true; ${\text{f}}\left( {\text{x}} \right)_{{\text{i}}}$, model output for input x; ${\text{BCE}}$, binary cross entropy.

Simultaneously, this form of the equation prevents the weighting factors from converging to zero or becoming too large.

These two methods proposed are conceptually different; nevertheless, the weighted binary cross-entropy function remains the loss function used for the calculation in both cases. The differences lie not in the type of loss function itself but in how the losses in predictions provided by each task are considered.

Notably the binary cross entropy loss functions we implement for each task considering the ratio of different classes for better imbalance and give the suggestion to network on which cases should give more importance. That means we trained our network using a balanced cross entropy.

### Software

All implementations in this work, including model development, data preprocessing, data analysis, and chemical encoding, were carried out using Python packages. The environment, along with all dependencies, is documented in a GitHub repository, where both the model and data are available. Model development and validation were performed using scikit-learn (version 1.4.0), and Mordred descriptors were calculated using the Mordred Python package (version 1.2.0).

## Results

### Applicability domain

Using the fixed data splitting defined as defined in Sect. “Internal and External Validation”, we calculated the applicability domain (AD), that is the part of the chemical space in which predictions can be considered reliable [48, 49]. For this purpose, we used an Unsupervised Outlier Detection approach and the Local Outlier Factor (LOF), which is a method that evaluates how isolated the object is with respect to the surrounding neighborhood. LOF measures the local deviation of the density of a given sample with respect to its neighbors’ molecules and then marks the sample as out of domain if the local density is substantially lower than those of its neighbors [50]. We performed this analysis using all encoders under study, except for the custom NLP embedding as they require a trained model.

We initially considered a chemical to be out-of-domain if it was classified as such by all encoders. When evaluating each encoder individually, out of the 2,203 chemicals in the holdout set, 170 (7.7%) were identified as out-of-domain by the Molecular Descriptors (MD), 16 (0.7%) by the Morgan Fingerprint, 3 (0.1%) by the CDDD encoder, and none by the ChemBERTa encoder. Based on these results, no chemical in the holdout dataset was classified as out-of-domain by all encoders, and thus, none should be considered out-of-domain. However, we explored potential performance improvements by applying a more restrictive applicability domain (AD), removing any chemicals identified as out-of-domain by at least one encoder. Applying this restriction resulted in the removal of 189 molecules (8.5%) from the holdout set. Model performance appears to improve slightly for certain tasks. The most significant improvement is observed in tasks related to the inhibition of mitochondrial complexes, where an increase in specificity was noted. The results of comparison between different ways to define AD are reported in Additional file 1: Sect. 5.

Notably, in literature, there are no well-established approaches specifically tailored for multitask models. However, it is important to recognize that one of the main strengths of multitask learning is its potential to broaden the applicability domain. This is because the prediction space is not limited to the data available for a single endpoint; rather, data from all task contribute to shaping the model’s representation space and, consequently, the AD. The data projection in chemicals space and further details regarding data distribution are reported in Additional file 1: Sect. 2.

### Baseline models

In Table 2 the performance of the best baseline model developed for each task is reported. In most cases, the performance of the models was strongly influenced by the class imbalance. For instance, considering mitochondrial dysfunction during internal validation, the sensitivity of the model is below 0.5, indicating that its ability to recognize active compounds is poor. This reflects the strong bias induced by the dataset, where only 23% of the data for mitochondrial dysfunction is active. This behavior is very common through endpoints.

**Table 2:** Baseline model results. The mean results of 5-fold cross-validation are reported, with the 95% confidence interval calculated using the t-student distribution. The results of holdout set are also reported.

Endpoints	Encoder	Holdout set			5-fold cross validation		
		Balanced accuracy	Sensitivity	Specificity	Balanced accuracy	Sensitivity	Specificity
Apical Cardiotoxicity	MD	0.66	0.95	0.37	0.61 ± 0.06	0.92 ± 0.03	0.29 ± 0.12
Aryl Hydrocarbon Receptor	CDDD	0.79	0.90	0.69	0.76 ± 0.07	0.97 ± 0.03	0.54 ± 0.13
Cardiomyocyte Myocardial Injury	MD	0.78	0.64	0.93	0.73 ± 0.03	0.55 ± 0.05	0.91 ± 0.02
Change Action Potential	CDDD	0.64	0.33	0.94	0.62 ± 0.07	0.27 ± 0.12	0.96 ± 0.04
Change in Inotropy	CDDD	0.64	0.30	0.98	0.58 ± 0.05	0.19 ± 0.09	0.97 ± 0.02
Change in Vasoactivity	MD	0.64	0.33	0.96	0.65 ± 0.02	0.34 ± 0.04	0.97 ± 0.01
Endothelial Injury Coagulation	CDDD	0.75	0.65	0.85	0.73 ± 0.01	0.64 ± 0.04	0.82 ± 0.04
hERG Channels Inhibitors	Morgan	0.80	0.80	0.80	0.79 ± 0.01	0.79 ± 0.02	0.79 ± 0.02
Increase Mitochondrial Dysfunction	MD	0.73	0.52	0.94	0.69 ± 0.02	0.44 ± 0.05	0.94 ± 0.01
Inhibition Mitochondrial Complexes	CDDD	0.62	1.00	0.25	0.54 ± 0.05	0.94 ± 0.05	0.14 ± 0.11
Oxidative Stress	MD	0.61	0.26	0.96	0.60 ± 0.05	0.25 ± 0.08	0.95 ± 0.05
Valvular Injury Proliferation	CDDD	0.65	0.41	0.89	0.63 ± 0.05	0.31 ± 0.12	0.95 ± 0.05

External validation performance is also reported to assess the model's ability to generalize to unseen data. The test performance reflects the same issue observed in cross validation.

As reported in Table 2, most baseline models struggle to generalize well when the class imbalance issue is not addressed. Only four baseline models achieved satisfactory performance in both internal and external validation. The models for Aryl hydrocarbon receptor binding and Cardiomyocyte myocardial injury perform relatively well in both internal and external validation, but with notable specificity or sensitivity limitations. The hERG channel inhibition and Endothelial injury and coagulation models perform well across all metrics, likely due to their more balanced datasets (51% and 43% active compounds, respectively), which reduces the performance bias.

### Multitask neural network: mixture of expert model

The best-developed single-encoder multitask model turned out to be the one based on the CDDD descriptors, as reported in Table 3. The CDDD MTNN, trained using weight loss, achieved satisfactory and consistent results both in cross-validation and in assessing chemicals in the holdout set, slightly outperforming the other single-encoder models.

**Table 3:** Best overall multitask model results without consider single endpoint performance. All true positives (TP), true negatives (TN), false positives (FP), and false negatives (FN) are aggregated across all tasks, and performance metrics are calculated based on these combined predictions to assess the global effectiveness of the multitask models. Results from 10-fold cross-validation and external validation are reported

	Holdout set			10-fold cross validation		
	Balanced accuracy	Sensitivity	Specificity	Balanced accuracy	Sensitivity	Specificity
CDDD weightedloss	0.76	0.75	0.76	0.75 ± 0.01	0.73 ± 0.03	0.76 ± 0.01
ChemBERTa singleloss	0.71	0.71	0.71	0.69 ± 0.01	0.68 ± 0.03	0.70 ± 0.03
MDs singleloss	0.74	0.78	0.70	0.68 ± 0.02	0.61 ± 0.10	0.76 ± 0.06
NLP custom singleloss	0.73	0.74	0.71	0.71 ± 0.02	0.70 ± 0.02	0.73 ± 0.02
Morgan weightedloss	0.74	0.75	0.73	0.71 ± 0.01	0.66 ± 0.02	0.75 ± 0.04

The CDDD MTNN outperforms most single-task models, which highlights the advantages of using a multi-task network. Further details of the results of single task encoder with the comparison on each task with machine learning model are reported in Additional file 1: Sect. 4..

**Fig. 4:**
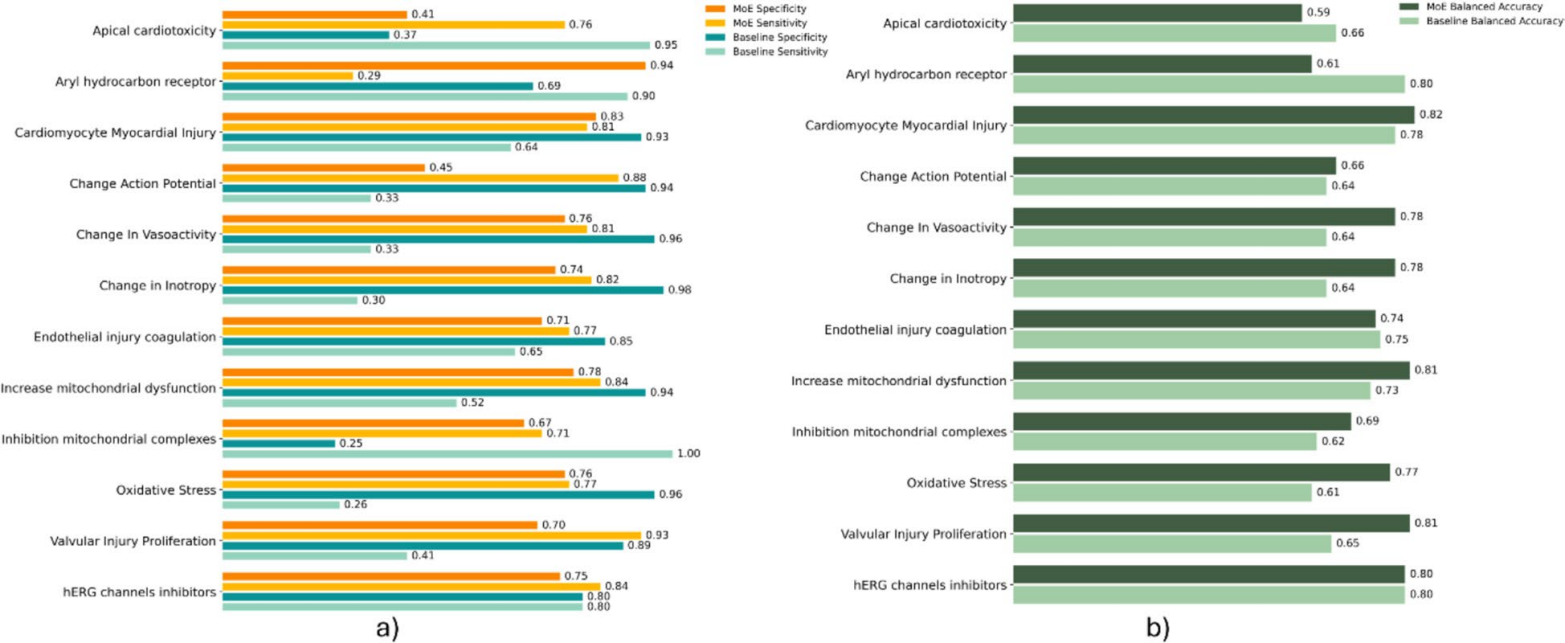
**a** Comparison of Sensitivity and Specificity between the performance of baseline models and the MoE on holdout set. **b** Comparison of balanced accuracy between the performance of baseline models and the MoE on holdout set

Based on the results from the baseline models and the single-encoder MTNN, we chose to combine the two best-performing encoders, which individually demonstrated strong performance. We then selected MDs and CDDD descriptors to develop the branches for MoE. The results obtained for the MoE are reported in Fig. 4 in comparison with the baseline RF.

Notably, for many endpoints where baseline models did not perform well, especially when strongly biased data affects the model's ability to recognize both activity classes, the MoE instead achieves more balanced predictive performances in cross validation and in holdout set. Indeed, the gap between sensitivity and specificity was significantly reduced for most of the endpoints under evaluation compared to the baseline results. As results, the mean absolute difference between sensitivity and specificity decreased from 0.46 in the baseline models (average value) to 0.17 for MoE, and the average balanced accuracy across endpoints increased from 0.69 to 0.74, demonstrating how the multitask effectively generalizes predictions to compounds belonging to different classes. To further compare the results, Sect. 4 of the Additional file 1 includes Table 3, which reports the values for each endpoint for the MoE and baseline models across specific metrics.

### Gate analysis

To extend our study, we explored a MoE using all encoders from the baseline models and compared our manual encoder selection with the gating mechanism’s weighting. Using all available data, we conducted 10-fold cross-validation, rebuilding the model each time, updating the gate values, and reporting the mean with a 95% confidence interval (*t*-student test, alpha = 0.05).

We reported the results only for the two endpoints for which the baseline models performed satisfactorily—hERG channel inhibitors, and endothelial injury and coagulation—since performance on other endpoints was generally too low.

The best overall baseline model is the hERG channel model, where Random Forest performs best with the Morgan fingerprints. This aligns with MoE gate weights, which prioritize the Morgan branch (87 ± 5%), followed by CDDD (8 ± 4%), and MDs/ChemBERTa (3 ± 1% each) branches. A similar trend is seen for endothelial injury and coagulation, where the best baseline model and MoE gate both highlight CDDD as the key branch (38 ± 9%).

### Real case scenario

To further validate our MoE models, we decided to assess substances collected by Krishna et al. in *High-Throughput Screening to Identify Chemical Cardiotoxic Pote*ntial [5], where their cardiotoxic effects reported in the literature is provided. This manually curated list of chemicals appears to be an ideal external test set for evaluating our method in a real-case scenario. For each chemical in the list, its positive or negative activity in in-vitro, in-vivo and in human epidemiology testing is reported, providing an additional layer of detail for assessing model performance. Additionally, the classification of each chemical is provided such as drugs, environmental, food additive and others. We decided to maintain three major classes: "Drugs" and "Environmental", as defined in the cited work, while all other chemicals were grouped into the "Other" category. The SMILES representation of each chemical underwent the same preprocessing steps used for the chemicals in the training dataset. After preprocessing, 225 out of the original 278 chemicals were retained. From those that passed preprocessing, we selected only the chemicals for which no experimental endpoint values were present in our dataset, retaining in the end 41 unique chemicals as our final real-case scenario test set.

**Fig. 5:**
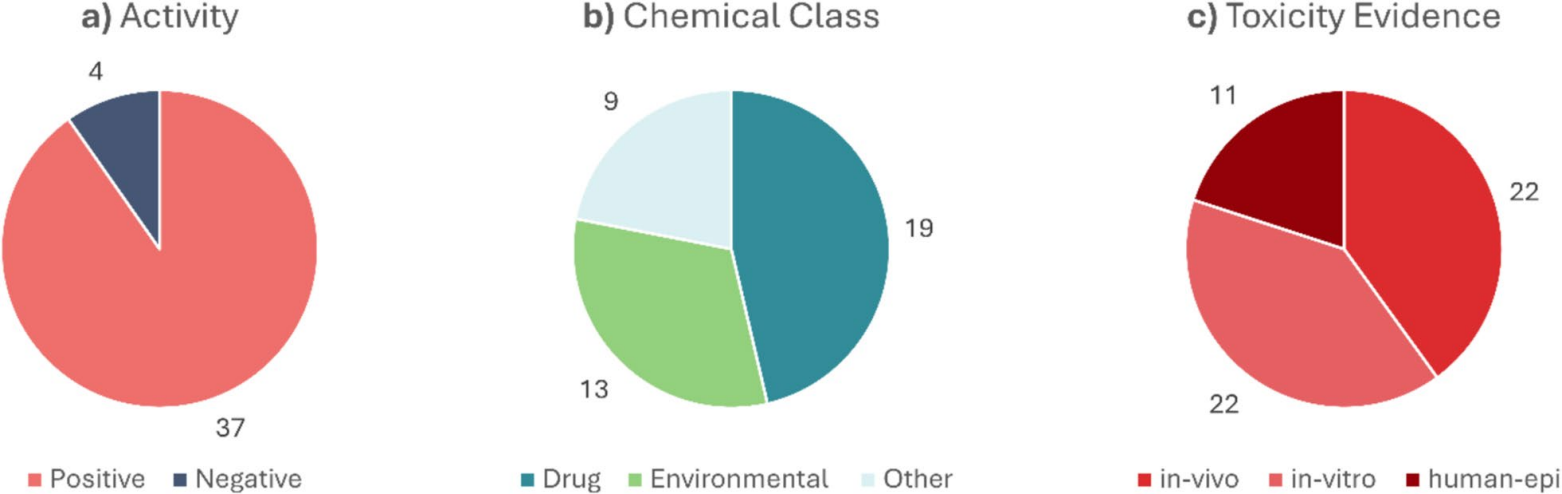
Pie charts representing the distribution of chemicals collected for the real case scenario. **a** Activity distribution, where "positive" refers to chemicals with cardiotoxicity evidence. **b** Distribution of chemicals across different classes. **c** Types of evidence found in the literature. A single chemical can have multiple types of evidence, meaning we may have information about its activity in vitro, in vivo, and/or in humans

Unfortunately, the list of chemicals reported in Krishna et al. is almost entirely composed of chemicals that exhibit toxic side effects. This provides us with a real-world case scenario that is strongly unbalanced for evaluation. Indeed, on 41 unique chemicals 37 are positive, that means 90% of data are active. However, it still serves as a useful means to assess how well our model can recognize truly toxic data. The data distribution for each activity class, each chemical class type, and whether the activity is based on in-vitro, in-vivo, or human epidemiological levels is presented in Fig. 5 with a corresponding pie chart.

Table 4 reports the sensitivity results of the MoE models developed in this work, as this metric appears to be the most suitable for evaluating the model’s ability to specifically recognize active chemicals.

**Table 4:** MoE results in predicting cardiotoxicity effects of chemicals belonging to the external test set

Evidence	Chemical class	Sensitivity	No.chemicals	No. positive
In-vitro	All	0.89	22	18
	Other	0.67	7	3
	Environmental	1.00	6	6
	Drug	0.89	9	9
In-vivo	All	0.81	22	21
	Other	0.33	4	3
	Environmental	1.00	8	8
	Drug	0.80	10	10
Human-epi	All	0.73	11	11
	Other	0.00	1	1
	Environmental	0.33	3	3
	Drug	1.00	7	7

A chemical is defined as active by the MoE model for in-vitro data if it is active in at least one of the endpoints trained on in-vitro tests. This means that the Apical Cardiotoxicity assessment is not considered when labeling in-vitro activity predictions.

For in-vivo and human data, a chemical is defined as active by the model’s prediction if it is classified as toxic in at least one model task including the Apical Cardiotoxicity. Considering the sensitivity values obtained, it is evident that the MoE model effectively recognizes and predicts potential cardiotoxic chemicals belonging to both drug and environmental classes.

## Discussion and conclusions

In our work, we conducted an extensive study to explore the potential applications of multitask learning (MTL) in toxicology, specifically for cardiotoxicity. We developed a series of baseline machine learning (ML) models as a starting point, which served as references to more complex approaches.

We encoded chemical information in multiple ways, ranging from traditional methods such as Morgan fingerprints and molecular descriptors to more recent NLP-based approaches, including ChemBERTa and CDDD encodings. For most of the evaluated endpoints, the best approaches for encoding chemical information were classical molecular descriptors (MDs) and CDDD. Both achieved the highest performance in baseline models, and CDDD demonstrated particularly promising results in capturing information useful for multitask networks. In fact, the best multitask model developed using a single encoder was based on CDDD. To enhance model performance, we implemented MoE architecture, which is considered by the authors the most suitable for managing feature injections. We designed two separate branches to handle encoded information from CDDD descriptors and MDs, improving generalizability over the single-encoder model. This architecture achieved a global balanced accuracy of 78% and an MCC of 0.54, calculated by aggregating true positives, true negatives, false positives, and false negatives across all endpoints in the multitask holdout set. Furthermore, analyzing the MoE gating layer allowed us to estimate the relative importance of each branch, making the network a valuable tool for identifying the most effective method for encoding chemical information.

The developed MLT aims to provide a more comprehensive understanding of cardiotoxicity caused by small molecules across multiple classes, including drugs, pesticides, industrial chemicals, and food additives. By considering multiple endpoints the model learns various toxicity effects simultaneously and identifies potential additive interactions that could lead to higher toxicity risks. Using data from MIEs (Molecular Initiating Events) and KEs (Key Events), the model improves predictions of MOA (Mechanisms of Action) effects, apical cardiotoxicity, and vice versa. The model’s performance aligns with this reasoning, as the multitask model outperformed the single-task model in nearly every task, effectively recognizing both activity classes for each task and thus increasing the balanced accuracy performance.

The results we achieved are promising, especially considering that there are several next steps that can further improve the model’s significance and performance. The mechanisms underlying cardiotoxicity are multifactorial, and our current model coverage remains limited. Important aspects like calcium cycling and various receptor-mediated pathways could be explored in more detail and additional mechanisms could be added during model training.

In our current work, we have aimed to extend the coverage of cardiotoxicity mechanisms by leveraging higher-level toxicity information, including known drug side effects in humans and their MOAs. These features allow us to capture a broad range of cardiotoxic effects. To enhance the completeness of our dataset and provide more mechanistic detail, we further integrated several well-characterized molecular MIEs and KEs, as described in the AOP network for cardiotoxicity [6–8, 15]. These include mitochondrial dysfunction and oxidative stress, which are also relevant to other types of toxicity. Although this data integration substantially improves the mechanistic coverage of our approach, additional refinement is possible through the inclusion of further relevant pathways and emerging toxicity data.

Notably, a considerable number of in-silico models related to cardiotoxicity can be found in the literature. Most of these focus on specific toxicity endpoints, such as mitochondrial dysfunction, hERG (human Ether-à-go-go-Related Gene) potassium channels, Nav1.5 sodium channels, Cav1.2 calcium channels, oxidative stress, and Aryl Hydrocarbon Receptor activity [31, 51–58]. This indicates that the literature already provides a wide coverage of cardiotoxicity-related modeling for small molecules. However, integrating multiple models to cover a wide range of cardiotoxicity events would be extremely time-consuming and resource-intensive. This is due to the need for multiple experts with different backgrounds to manage models implemented in various programming languages or requiring different execution modes (e.g., batch versus single-molecule processing). In contrast, our unified framework simplifies the evaluation process by allowing the prediction of multiple endpoints using a single model. This makes the assessment of the cardiotoxic potential of small molecules more efficient, improves usability, reduces the burden of tool integration, and supports broader applicability in real-world screening and regulatory contexts.

The performance achieved by our method is comparable with state-of-the-art results for the tasks under evaluation and, for specific endpoints, outperform those reported in the literature, although a direct comparison is not straightforward due to differences in the underlying datasets.

For instance, in the case of oxidative stress, our model achieved a specificity of 0.76 and a sensitivity of 0.77 on the holdout set. In comparison, traditional machine learning models reported in the literature have achieved, at best, a specificity of 0.76 and a sensitivity of 0.68, using oversampling techniques to enhance performance [30]. For mitochondrial dysfunction, our MTL model achieved a specificity of 0.78 and a sensitivity of 0.84 on the holdout set, which outperform recent models results reported in the literature. One study reported a sensitivity of 0.79 and a specificity of 0.69 [56], while another reported slightly lower performance, with a sensitivity of 0.74 and a specificity of 0.67 [55]. Additionally, other advanced neural networks models developed for predicting mitochondrial dysfunction, such as the one presented in *Predicting the Mitochondrial Toxicity of Small Molecules: Insights from Mechanistic Assays and Cell Painting Data* [31], reported a sensitivity of 0.73.

Regarding mitochondrial complex inhibition, considered a key mechanism in respiratory chain disruption, our MTL model achieved a sensitivity of 0.67 and a specificity of 0.71. These results are comparable to those in the literature, where models reached a sensitivity of 0.59 (± 0.10) and a specificity of 0.78 (± 0.05) [31]. For hERG inhibition instead, our model achieved a specificity of 0.75 and a sensitivity of 0.84 on holdout set, outperforming several literature-reported models. As instance, the models developed in “Ligand-based prediction of hERG-mediated cardiotoxicity based on the integration of different machine learning techniques” [21] achieve a sensitivity of 0.67 and a specificity of 0.76 on external set or CUPID platform [51] report a sensitivity 0.664 ± 0.008 and specificity 0.647 ± 0.008, while other, such as the CardioToxNet model hosted on OCHEM [59] report a specificity of 0.79 and a sensitivity of 0.83 [52].

These endpoints represent some of the most extensively studied Molecular Initiating Events (MIEs) and Key Events (KEs), not only in the context of cardiotoxicity but also in relation to other toxicity outcomes such as steatosis and cholestasis.

To conclude and further test our approach we applied MoE models to predict an external test set consisting of chemicals known from literature to induce cardiotoxicity effects. This data is highly unbalanced, as 90% of the chemicals collected from the literature are associated with cardiotoxicity. This represents a significant limitation for genuinely validating the model on an external dataset. However, the results from the assessment highlighted that the model demonstrates strong capability in recognizing active chemicals, achieving high sensitivity performance. This means that the MoE could be an important candidate as a first-tier component in advanced New Approach Methodologies to identify chemicals that could be further tested, which was ultimately our initial aim.

## Supplementary Information

This file provides extended methodological details, including dataset construction and preprocessing (from sources such as NIH ICE, ChEMBL, and DICTrank), SMILES standardization, and endpoint-specific class distributions. It includes chemical space visualizations (t-SNE plots), model comparison figures, and additional tables to support and expand upon the findings reported in the main text. The file also describes the architecture and implementation of the multitask and mixture-of-experts (MoE) models, encoder configurations, and performance evaluation metrics across endpoints

**Additional file 1.**

## Data Availability

Data is provided within the manuscript. The data used to develop the models are freely accessible at: https://github.com/EdoardoVigano/MoECardiotoxicity. Additionally, collected data and developed models are available at https://platform.alternative-project.eu/, along with a ready-to-use API. The multitask model is also available on VEGA software downloadable in VEGAHUB https://www.vegahub.eu/.
