# Supplementary material for: Mixture of experts for multitask learning in cardiotoxicity assessment
Source: J Cheminform. 2025 Aug 29;17:135. doi: 10.1186/s13321-025-01072-7 (PMC12395882; doi:10.1186/s13321-025-01072-7)
Supplement: Supplementary file 1 — Additional file 1. [file 13321_2025_1072_MOESM1_ESM.docx]

# Supplementary Materials

1. Dataset and data curation

Two datasets were used in this study: NIH ICE database and ChEMBL. The ICE database provides curated data intended to support the development and evaluation of new, revised, and alternative methods and is developed by NICEATM, ICCVAM and their partners. ChEMBL is a manually curated database for small, drug-like molecules that includes information about their structures, molecular properties, measurements from various assays, and genomic data.

The chemicals were retrieved using CAS numbers, which are unique identifiers assigned to each molecule. To obtain the Simplified Molecular Input Line Entry System (SMILES), we used an in-house software (<https://github.com/EdoardoVigano/Chemical-Resolver>), which can query the API service of reliable databases such as ChEMBL and PubChem. The obtained SMILES strings were then standardized following a protocol [39,40] that involves removing organometallic and inorganic compounds, eliminating chemicals with structural inconsistencies and mixtures, and removing the stereochemistry information. During curation, we removed molecules with extreme physicochemical properties—for example, compounds with logP values < –5 or > 7, molecular weights < 12 u or > 600 u, and those with fewer than 3 or more than 50 heavy atoms. These constraints helped define the chemical space in which our model operates, contributing to a more clearly defined applicability domain. The SMILES obtained were then canonized, and duplicate structures were removed.

Each chemical was then labeled as active for a given biological target if at least one of the assays for that target had a positive label, and inactive otherwise. To determine positivity in a test, the procedure described in the *User's Guide for Accessing and Interpreting ToxCast™*  [41] is followed. Raw data provided by a vendor or laboratory undergoes processing, indexing, transformation, and normalization using standardized methods. Subsequently, the concentration-response data are subjected to modelling through three selected models (constant, Hill, and gain-loss).  If any models fit sufficiently, the chemical–assay pair is considered ‘active’ (hit call = active); otherwise, the final hit call is ‘inactive’. All information about assays is available in the ICE database (https://ice.ntp.niehs.nih.gov/).

Regarding the ChEMBL database, the procedure for obtaining the final data varies for each endpoint as described below in paragraph 1.1, 1.2 and 1.3.

The final dataset consists of 14,688 unique chemicals. Notably, the datasets are unbalanced for almost all endpoints, and the statistics on dataset composition are reported in the following paragraphs.

1. Mode Of Actions (MOAs)

The following cardiotoxicity-related datasets were retrieved from the ICE database: “Change Action Potential”, “Cardiomyocyte Myocardial Injury”, “Change in Inotropy”, “Endothelial injury coagulation”, “Valvular injury proliferation”, and Change in Vasoactivity”. The descriptions of data collected for MOAs are reported in Table 1.

Table 1: Summary of data for MOA with information, total number of chemicals, and percentages of active and inactive compounds.

|  | #Compounds | #active | #inactive | %active | %inactive |
| --- | --- | --- | --- | --- | --- |
| Cardiomyocyte Myocardial injury | 5306 | 1558 | 3748 | 29 | 71 |
| Change Action Potential | 415 | 111 | 304 | 27 | 73 |
| Change In Inotropy | 922 | 213 | 709 | 23 | 77 |
| Change in Vasoactivity | 4955 | 1009 | 3946 | 20 | 80 |
| Endothelial Injury Coagulation | 5350 | 2290 | 3060 | 43 | 57 |
| Valvular Injury Proliferation | 267 | 91 | 176 | 34 | 66 |

The datasets obtained for MOAs are almost all strongly unbalanced. For 4 out of 6, the percentage of inactive compounds exceeds 70% of the total. Specifically, for changes in vasoactivity, 80% of the data related to this endpoint are classified as inactive. The “Endothelial injury coagulation” was the only balanced dataset.

1. Molecular Initiative Events (MIEs) and Key Events (KEs)

Data for specific MIE and KEs, such as inhibition of mitochondrial complexes, increased mitochondrial dysfunction, or oxidative stress are found in ICE database. To further expand our dataset and cover additional MIEs we used the ChEMBL database (version 28) to retrieve data for two additional targets: hERG channel blockade (ChEMBL240) and aryl hydrocarbon receptor (AHR) interaction (ChEMBL3201).

The “activity comment” and “pChEMBL” fields were used to define a threshold for binary classification. Data marked as inconclusive or with a validity comment of *&apos;not* outside typical *range&apos;* were discarded. If the activity comment was not present a compound was labelled as active if the pChEMBL value was above 0.5.

For the aryl hydrocarbon receptor, after the curation and standardization process, 328 out of 1071 initially retrieved chemicals were added to the dataset.

Regarding hERG channel blockade, the retrieved data comprised a total of 17,952 activity entries based on the Target ID (ChEMBL240) assigned to the hERG channel. To ensure data validity, the database was filtered to retain only entries matching the following criteria: (1) annotated exclusively with IC50 measurements (11,144 entries), (2) referring to assays conducted on human targets (“target_organism” = “Homo sapiens”), (3) marked as direct binding (“assay_type” = “B”), and (4) free of warnings in the “data_validity_comment” field. Each IC50 value was converted from molar concentration (M) to pIC50 (–log IC50), and compounds without pIC50 values, but already marked as inactive in the ChEMBL repository, were also considered. Consistent with the literature [17–24] we defined the activity of hERG-DB as hERG blockers (ACT) with an IC50 ≤ 1 µM (pIC50 ≤ 6), compounds with moderate hERG blocker potential (5 < pIC50 ≤ 6), and hERG non-blockers (INA) having IC50 values > 10 µM (pIC50 > 5). In our dataset, we set a threshold of pIC50 > 6 to define inactivity. This approach allows us to be more conservative and classify moderate hERG blockers as active as well. The SMILES were curated in the same way as described previously.

The data collected for the MIEs is reported in table 2.

Table 2: Summary of data for KEs and MIEs with information, total number of chemicals, and percentages of active and inactive compounds

|  | AOP | #Compounds | #active | #inactive | %active | %inactive |
| --- | --- | --- | --- | --- | --- | --- |
| Aryl hydrocarbon receptor | MIE | 328 | 211 | 117 | 64 | 36 |
| hERG channels inhibitors | MIE | 8461 | 4294 | 4167 | 51 | 49 |
| Inhibition mitochondrial complexes | MIE | 232 | 184 | 48 | 79 | 21 |
| Increase mitochondrial dysfunction | KE | 4964 | 1131 | 3833 | 23 | 77 |
| Oxidative Stress | KE | 640 | 193 | 447 | 30 | 70 |

1. Apical Cardiotoxicity Drugs Side Effects (DICTrank)

The last data source we used is a dataset of drugs annotated with ranked drug-induced cardiotoxicity risk in humans. These data were collected by utilizing labelling documents for FDA (U.S. Food and Drug Administration)-approved drugs. As claimed by the authors, this is the largest dataset of drugs annotated with ranked DICT risk in humans (DICTrank) [38].

DICTrank categorizes drugs into four categories of DICT concerns by integrating DICT severity and labeling content. The dataset consists of 1318 drugs, classified as follows: Most-DICT-Concern (341), Less-DICT-Concern (528), No-DICT-Concern (343), and Ambiguous-DICT-Concern (106; lacking sufficient information in the labeling document to determine cardiotoxicity potential). The “ambiguous” entries were discarded, and the “Less-DICT-Concern” and “Most-DICT-Concern” entries were merged. After data curation and duplicate removal, we obtained 846 unique chemicals, of which 620 (73%) are classified as active. The remaining 226 chemicals are considered inactive, as no cardiotoxicity-related side effects have been reported for these drugs in the FDA report.

1. Data analysis

We represented the chemical space using the Morgan fingerprints, a method commonly used in QSAR for chemical representation, and the t-distributed Stochastic Neighbor Embedding (t-SNE) method, which is a nonlinear dimensionality reduction technique that transforms high-dimensional data to a low-dimensional space. During this transformation the local structure of the data is preserved so that similar points in the high-dimensional space remain close together in the projection space. The t-SNE projections, colored by experimental activity, are shown in Figure 1.


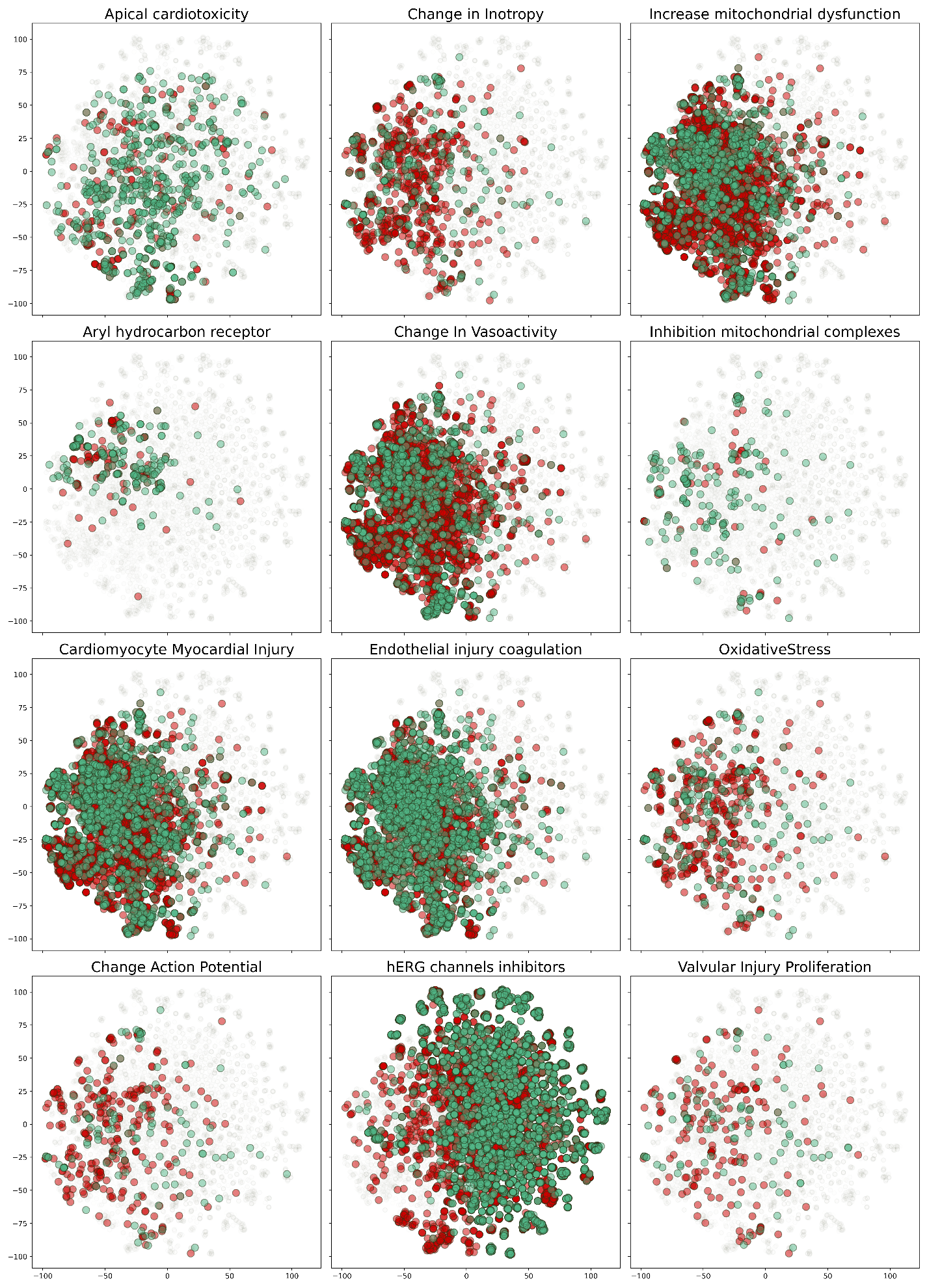


Figure 1: Projection of the entire dataset using t-SNE. Each plot shows the dataset distribution in t-SNE space, with chemical activity indicated by color: red for active compounds and green for inactive ones for the specific endpoint.

The uniformity of the molecular distribution varies between endpoints. For instance, molecules are well spread across the chemical space for most endpoints, including those with lower numerosity, which are still well distributed throughout the space despite being less represented. Only the aryl hydrocarbon receptor appears to have most of its tested data confined to a specific region of the chemical space. Differences in dataset distribution and numerosity are common in toxicity databases due to various reasons. For example, some endpoints are tested more frequently than others due to factors such as the simplicity of available in vitro tests, cost, and time constraints.

1. Model architecture details

After the input layer, three fully connected formed the shared representation of the multitask network. The final part of the architecture consisted of 12 towers, one for each endpoint, each containing two linear layers. A leaky ReLU activation function and a batch normalization layer are applied after each linear layer except the last one, as they enhance model performance by capturing nonlinear relationships and normalizing input values.

Regarding the NLP-based custom model, we implemented an embedding branch at the beginning of the network to embed the SMILES representation, something not required for the other encoders. From previous studies conducted on the mitochondrial dysfunction endpoint, we demonstrated that Natural Language Processing (NLP) methods are promising for predicting this fundamental KE event in the AOP network, which leads to cardiotoxic effects. Based on these findings, we initiated experiments using a custom NLP model for MTL. These approaches apply character/word embeddings directly to chemical notations as sequences of characters, without requiring additional preprocessing or intermediate steps. Tokenization is used to break the text into smaller units, or tokens, which may consist of single characters or groups of characters. We adopted the atomwise tokenizer for tokenization, as previous research identified it as the most promising approach. Network architecture to encode SMILES notation comprises several layers which are reported in Figure 2.


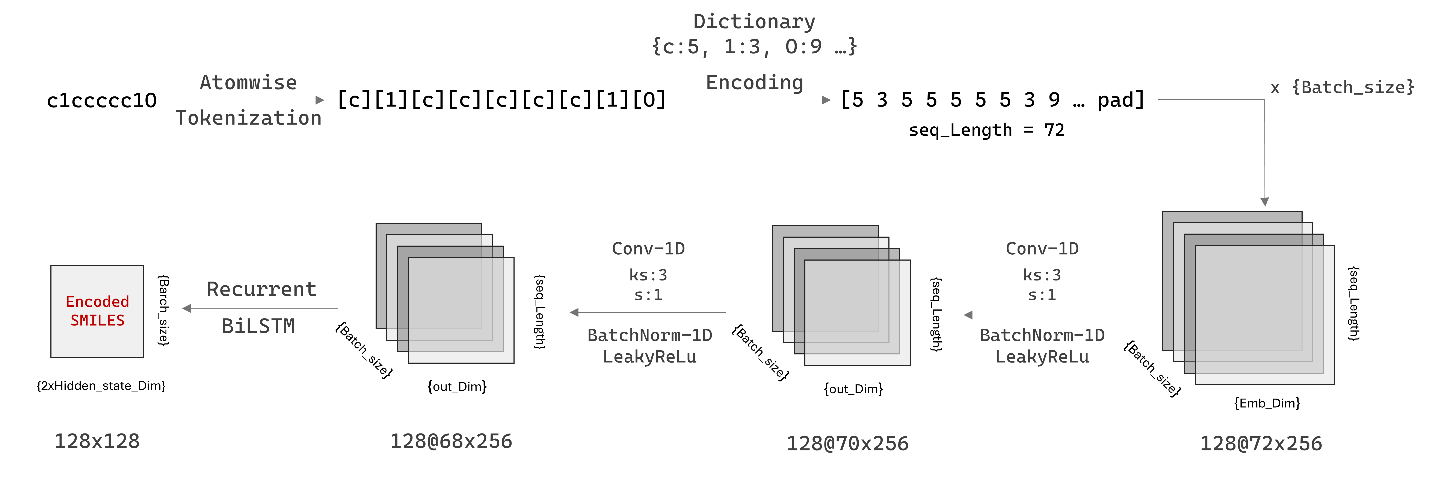
The initial block includes the Input Layer, followed by the Tokenizer Layer and the Embedding Layer, which encodes each tokenized character as a dense numerical array. Next, Convolutional Layers (Conv1D) perform convolution operations, capturing local patterns within the embedded chemical notation strings. The convolution results are then processed by Bidirectional Long Short-Term Memory (LSTM) layers, designed to capture contextual information from both preceding and succeeding tokens in the chemical notation strings. The hidden state of the LSTM represents the relevant features from the Bidirectional LSTM output and is used as input for the subsequent layers.

Figure 2: NLP custom embedding series of layers to encode SMILES notation.

This mechanism estimates which of the proposed encoders is the most suitable for further exploration and testing to assess a specific endpoint. Indeed, the relevance of each branch should differ depending on the endpoint. For this reason, we implemented the gate as a tensor with dimensions corresponding to (output dimension of each branch) × (number of branches) × (number of towers). This tensor performs a weighted sum of the encoded information from the branches, generating a comprehensive i-th array that serves as the input for the i-th tower.

1. Multitask Neural Network detailed comparison between Baseline Models, Single Encoder multitask network and MoE.

For many endpoints where baseline models did not perform well, especially when strongly biased data affects the model's ability to recognize both activity classes, the multitask model achieves more balanced predictive performances in cross validation and in external set. These endpoints include Cardiomyocyte Myocardial Injury, Changes in Action Potential, Changes in Inotropy, Changes in Vasoactivity, Increased Mitochondrial Dysfunction, Oxidative Stress, and Valvular Injury Proliferation. In contrast to the baseline models, the multitask model predicts the chemicals in a consistently well-balanced manner across these endpoints

The best model in general terms is the one that uses the CDDD encoder. That achieves higher performance in five out of seven metrics we selected to evaluate the models’ performance. Regarding instead of the single endpoint evaluation, we reported in Figure 3 the results for the best network overall in comparison with the baseline single task model developed.


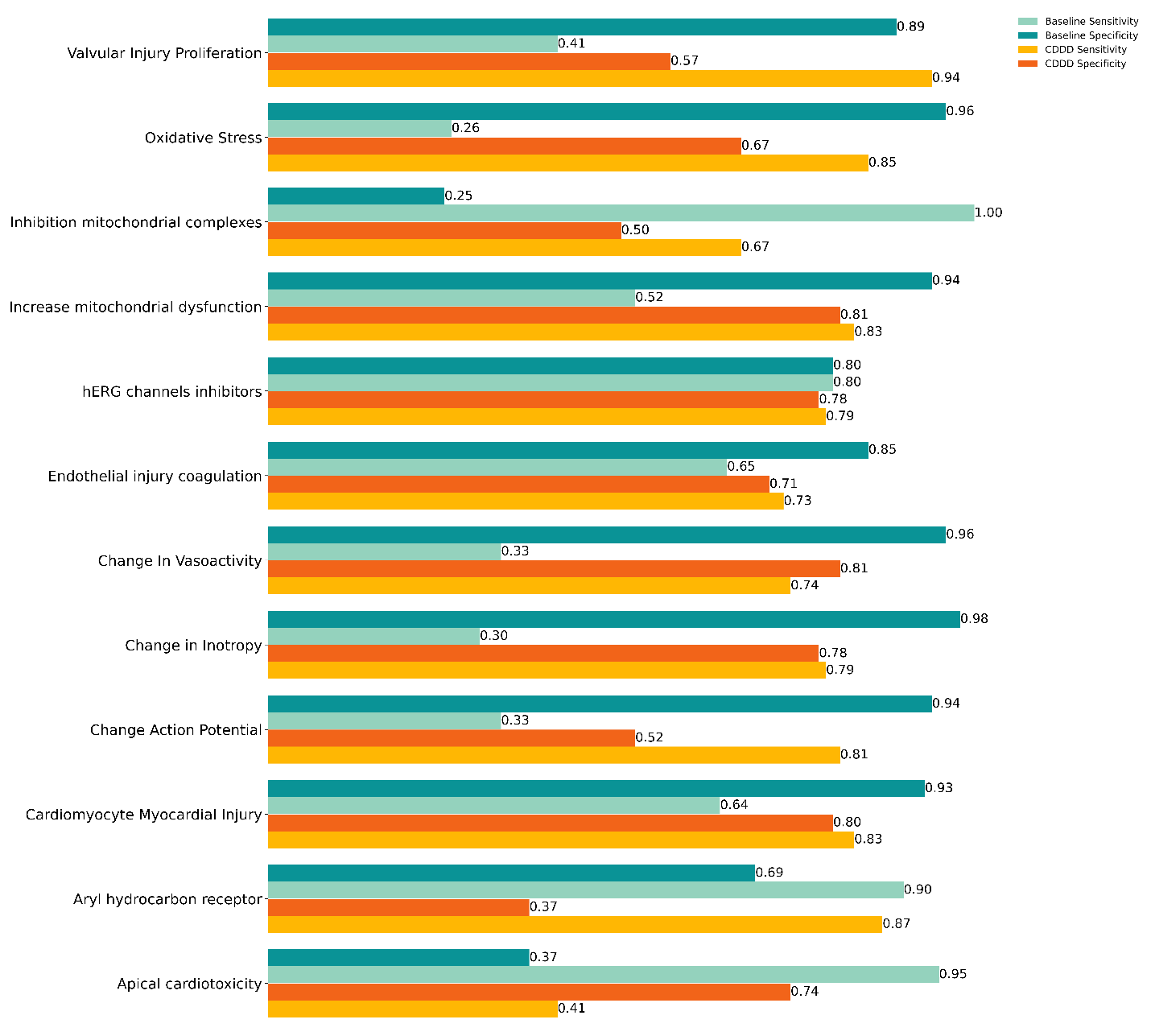


Figure 3: Comparison of Sensitivity and Specificity between the performance of Baseline models and the Multitask Single Encoder CDDD on holdout set.

The results on the holdout set for the developed MoE architecture are reported in Figure 4 where the MoE performances are compared with Multitask network with only CDDD encoder.


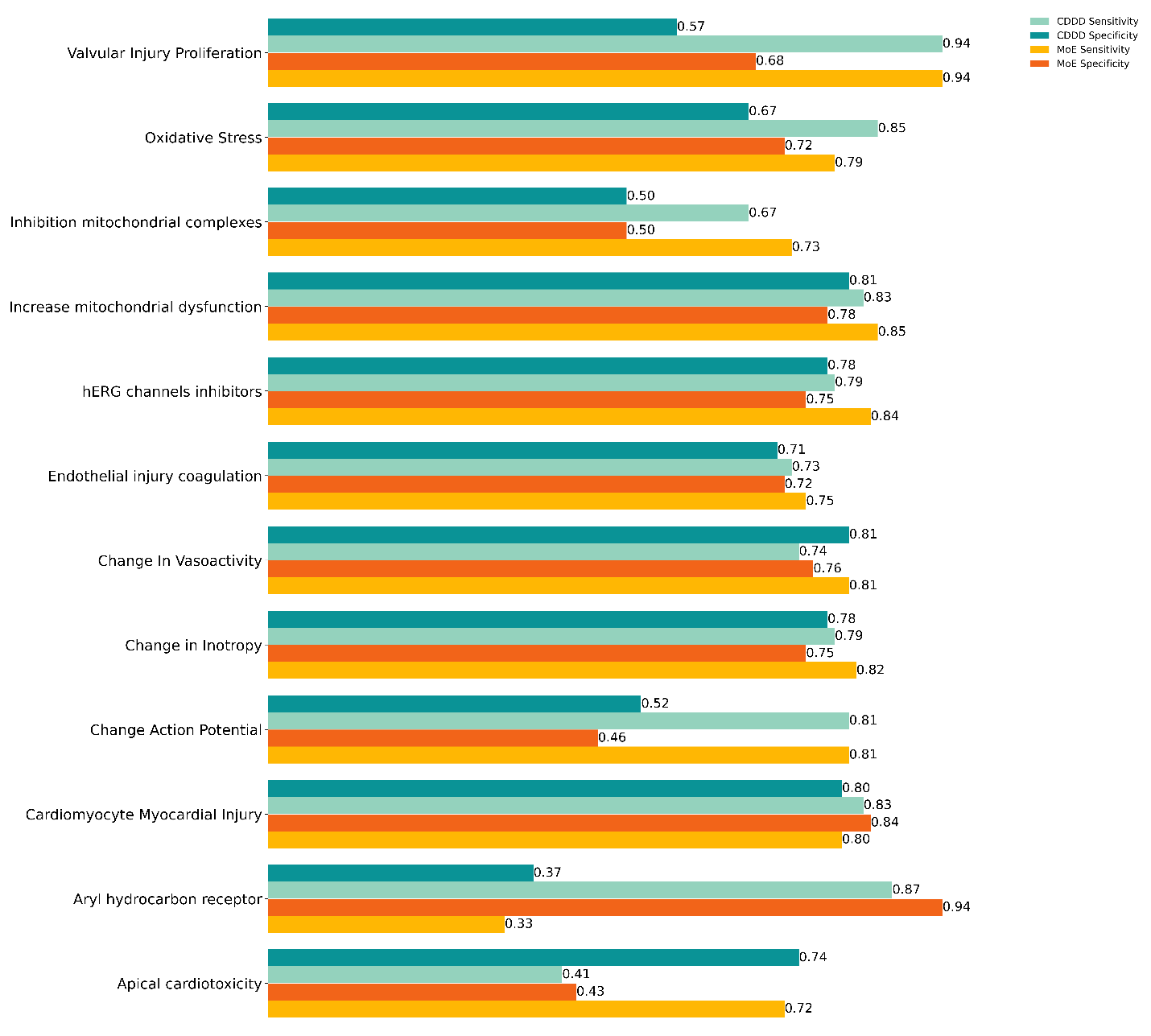


Figure 4: Comparison of Sensitivity and Specificity between the performance of Multitask Single Encoder CDDD and MoE CDDD-MDs on holdout set.

There are slight improvements across several of the endpoints under consideration, such as cardiomyocyte myocardial injury, changes in vasoactivity, endothelial injury and coagulation, hERG channel inhibition, mitochondrial complex inhibition, oxidative stress, and valvular injury proliferation. Considering the overall performance—meaning the balance between correct assessments and incorrect predictions across each endpoint—the results on the holdout set show that the MoE model outperforms the single-encoder CDDD model across various metrics, including Balanced Accuracy, Sensitivity, Specificity, MCC, and F1-Score. Specifically, the MoE model achieved a balanced accuracy of 0.78, sensitivity of 0.80, and F1-Score of 0.73, while the CDDD model reached a balanced accuracy of 0.77, sensitivity of 0.76, and F1-Score of 0.71. These improvements highlight the benefit of incorporating molecular descriptors as an additional branch in the MoE model. This allows the MoE model to extract a more comprehensive view of the molecular information compared to the NLP-based CDDD approach, enhancing its overall predictive performance.

The results indicate that the MoE model generalizes better from the training set, achieving superior performance on the holdout test set. This highlights the importance of providing the model with multiple sources of chemical information to enhance its overall learning capability.

To further compare the results, Table 3 reports the values for each endpoint for the MoE and Baseline models across specific metrics.

Table 3: Comparison of Baseline and MoE on each endpoint. The last row reports the mean values across endpoints for each metric.

| Endpoints | Baseline Balanced Accuracy | MoE Balanced Accuracy | Baseline Sensitivity | MoE Sensitivity | Baseline Specificity | MoE Specificity |
| --- | --- | --- | --- | --- | --- | --- |
| Apical cardiotoxicity | 0.66 | 0.59 | 0.95 | 0.76 | 0.37 | 0.41 |
| Aryl hydrocarbon receptor | 0.80 | 0.61 | 0.90 | 0.29 | 0.69 | 0.94 |
| Cardiomyocyte Myocardial Injury | 0.78 | 0.82 | 0.64 | 0.81 | 0.93 | 0.83 |
| Change Action Potential | 0.64 | 0.66 | 0.33 | 0.88 | 0.94 | 0.45 |
| Change in Inotropy | 0.64 | 0.78 | 0.30 | 0.82 | 0.98 | 0.74 |
| Change In Vasoactivity | 0.64 | 0.78 | 0.33 | 0.81 | 0.96 | 0.76 |
| Endothelial injury coagulation | 0.75 | 0.74 | 0.65 | 0.77 | 0.85 | 0.71 |
| hERG channels inhibitors | 0.80 | 0.80 | 0.80 | 0.84 | 0.80 | 0.75 |
| Increase mitochondrial dysfunction | 0.73 | 0.81 | 0.52 | 0.84 | 0.94 | 0.78 |
| Inhibition mitochondrial complexes | 0.62 | 0.69 | 1.00 | 0.71 | 0.25 | 0.67 |
| Oxidative Stress | 0.61 | 0.77 | 0.26 | 0.77 | 0.96 | 0.76 |
| Valvular Injury Proliferation | 0.65 | 0.81 | 0.41 | 0.93 | 0.89 | 0.70 |
| Average | **0.69** | **0.74** | **0.59** | **0.77** | **0.80** | **0.71** |

1. Applicability Domain and model performance

We considered applying a more restrictive applicability domain (AD) to explore potential improvements in model performance.

Instead of labeling a chemical as out-of-domain only if it was classified as such by all encoders, we chose to remove any chemical identified as out-of-domain by at least one encoder.


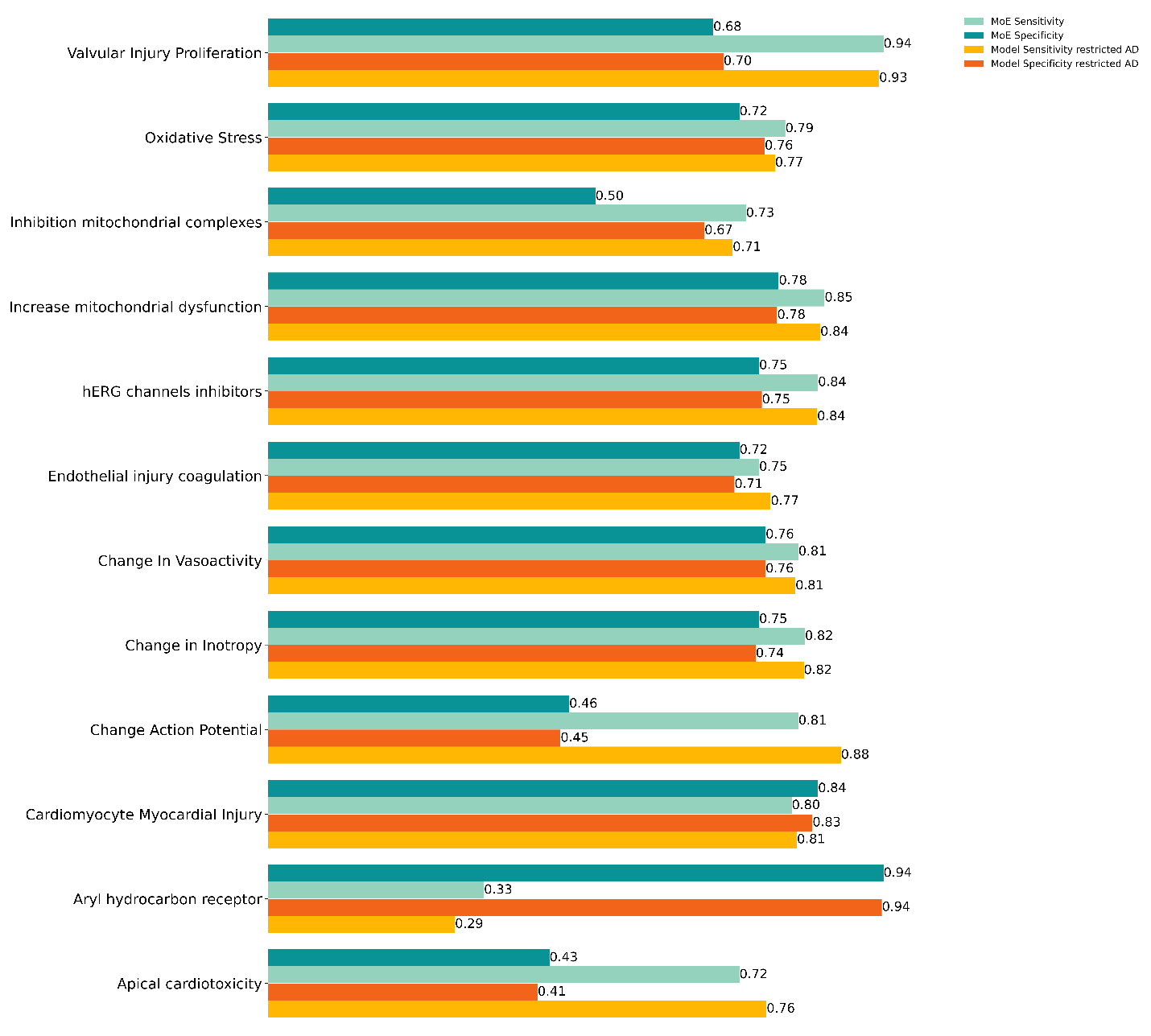


Figure 5: comparison of model performance for each task for extended and stricter AD.

Applying this restriction resulted in the removal of 189 molecules (8.5%) from the holdout set. Model performance appears to improve slightly for certain tasks. The most significant improvement is observed in tasks related to the inhibition of mitochondrial complexes, where an increase in specificity was noted. In general, the overall balanced accuracy increased from 0.73 to 0.74 under a restrictive applicability domain.
